# High Open Circuit Voltage Over 1 V Achieved in Tin‐Based Perovskite Solar Cells with a 2D/3D Vertical Heterojunction

**DOI:** 10.1002/advs.202200242

**Published:** 2022-04-22

**Authors:** Tianyue Wang, Hok‐Leung Loi, Jiupeng Cao, Zhaotong Qin, Zhiqiang Guan, Yang Xu, Haiyang Cheng, Mitch Guijun Li, Chun‐Sing Lee, Xinhui Lu, Feng Yan

**Affiliations:** ^1^ Department of Applied Physics The Hong Kong Polytechnic University Hung Hom Kowloon Hong Kong SAR 999077 P. R. China; ^2^ Department of Physics The Chinese University of Hong Kong Shatin Hong Kong SAR 999077 P. R. China; ^3^ Center of Super‐Diamond and Advanced Films (COSDAF) Department of Chemistry City University of Hong Kong Kowloon Tong Hong Kong SAR 999077 P. R. China; ^4^ Division of Integrative Systems and Design Department of Electronic and Computer Engineering The Hong Kong University of Science and Technology Clear Water Bay Kowloon Hong Kong SAR 999077 P. R. China; ^5^ Research Institute of Intelligent Wearable Systems The Hong Kong Polytechnic University Hung Hom Kowloon Hong Kong 999077 P. R. China

**Keywords:** 2D/3D, high open circuit voltage, tin‐based perovskite solar cell, vertical heterojunction

## Abstract

2D–3D mixed tin halide perovskites are outstanding candidate materials for lead‐free perovskite solar cells (PSCs) due to their improved stability and decreased trap density in comparison with their pure 3D counterparts. However, the mixture of multiple phases may lead to poor charge transfer across the films and limit the device efficiency. Here, a stacked quasi‐2D (down)–3D (top) double‐layered structure in perovskite films prepared via vacuum treatment is demonstrated, which can result in a planar bilayer heterojunction. In addition, it is found that the introduction of guanidinium thiocyanate (GuaSCN) additive can improve the crystallinity and carrier mobility in the 2D perovskite layer and passivate defects in the whole film, leading to a long carrier lifetime (>140 ns) in photoluminescence measurements. As a result, the PSCs show a high open circuit voltage (V_OC_) up to 1.01 V with a voltage loss of only 0.39 V, which represents the record values ever reported for tin‐based PSCs. The champion device exhibits a power conversion efficiency (PCE) of 13.79% with decent stability, retaining 90% of the initial PCE for 1200 h storage in N_2_‐filled glovebox.

## Graphical abstract

The type‐II 2D/3D vertical heterojunction is created in tin‐based perovskite films, which is favorable for charge transfer. Moreover, guanidinium thiocyanate additive is introduced to construct conducting channels for hole transfer in the 2D layer and reduce recombination loss in the whole films. The resultant solar cells show a record open circuit voltage of 1.01 V.

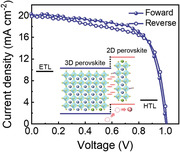

## 1 Introduction

Perovskite solar cells (PSCs) based on environmentally friendly lead (Pb)‐free perovskites have attracted much attention recently for the high potential in practical applications,^[^ ^1^, ^2^ ^]^ in which tin (Sn)‐based perovskites have been regarded as one of the most promising candidate materials for the devices. In 2014, the Snaith group and the Kanatzidis group independently reported MASnI_3_ and MASnI_3 ‒ x_Br_x_‐based PSCs fabricated on mesoporous TiO_2_ scaffolds with a power conversion efficiency (PCE) of 6.4% and 5.73%, respectively.^[^ ^3^, ^4^ ^]^ Later on, Yan's group reported inverted FASnI_3_ ‐based PSCs with a PCE of 6.22%.^[^ ^5^ ^]^ The initial achievements inspire the further development of this field. Recently, new records of over 14% PCE have been achieved for Sn‐based PSCs.^[^ ^6^, ^7^ ^]^ However, the performance of Sn‐based PSCs still lags far behind their Pb‐based counterparts although the two types of perovskite materials have comparable optoelectronic properties like suitable bandgaps and high enough carrier mobilities for solar cells.^[^ ^8^, ^9^, ^10^ ^]^

The major drawback of Sn‐based PSCs is their low open circuit voltages (*V*_OC_), which are normally between 0.2 and 0.7 V for devices with pure 3D perovskites,^[^ ^5^, ^11^, ^12^, ^13^, ^14^, ^15^, ^16^, ^17^, ^18^, ^19^ ^]^ due to high voltage losses induced by fast non‐radiative carrier recombination in the Sn‐based perovskite materials.^[^ ^20^, ^21^ ^]^ To the best of our knowledge, the carrier lifetimes in Sn‐based perovskites measured by photoluminescence (PL) spectroscopy are only several to tens of nanoseconds,^[^ ^6^, ^7^, ^22^, ^23^, ^24^ ^]^ which are much shorter than those of Pb‐based perovskites reported in literature.^[^ ^25^, ^26^ ^]^ The major reason can be attributed to the high‐level p‐type doping induced by the oxidation of Sn^2+^. Hence, various strategies, for example, perovskite grain boundary/surface functionalization,^[^ ^27^, ^28^, ^29^ ^]^ and compositional engineering^[^ ^30^, ^31^ ^]^ have been developed to suppress the oxidation of Sn^2+^ in the perovskite materials. The introduction of 2D/3D mixed perovskite structures has been recognized to be a most effective approach for passivating defects through the coordination between the 2D spacer and the uncoordinated Sn^2+^ and suppressing Sn^2+^ oxidation,^[^ ^32^, ^33^, ^34^, ^35^, ^36^, ^37^, ^38^ ^]^ and the record efficiency for Sn‐basd PSCs has been achieved by this method.^[^ ^6^, ^7^, ^39^ ^]^ Notably, *V*_OC_ was improved to 0.94 V in the devices based on PEA*_x_* FA_1−_ *_x_* SnI_3_ with a voltage loss of about 0.45V.^[^ ^39^ ^]^ However, the 2D perovskite phase has a wider bandgap than that of a 3D phase and may prohibit carrier transport in the perovskite films.^[^ ^40^, ^41^ ^]^ To solve this problem, one approach is to establish a 2D/3D stratified heterojunction that can enable charge separation at the interface,^[^ ^42^ ^]^ which however has not been observed in Sn‐based perovskites until now.

In this work, we found for the first time that vacuum treatment immediately after film coating can enable obvious 2D/3D phase stratification in Sn‐based perovskite films. We speculate that the quick evaporation of organic solvent from the top surface makes the 3D perovskite with lower solubility in the organic solvent solidified on the top and the more soluble 2D phase aggregated on the bottom. More importantly, the resultant 2D/3D heterojunction has a staggered gap (type II), which is favorable for charge separation in the device. Then, we introduce guanidinium thiocyanate (GuaSCN) to tune the electronic properties in the heterojunction as an additive. Interestingly, we find that GuaSCN additive can improve the crystallinity of 2D perovskite phase, increase its hole mobility, and passivate traps in the whole perovskite film with a prolonged carrier lifetime of over 140 ns characterized by PL measurements. Thanks to the synergistic effect of these efforts, the devices show a record *V*_OC_ of 1.01 V and a low voltage loss of 0.39 V, indicating that high *V*_OC_ loss of Sn‐based PSCs can be overcome by optimizing the 2D/3D heterojunctions.

## 2 Results and Discussion

**Figure 1:**
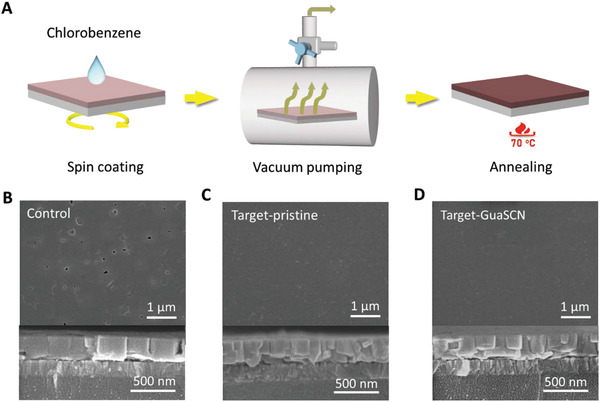
Formation of 2D/3D vertical heterojunction in the perovskite film via vacuum treatment. A) Schematic diagram of the vacuum assisted fabrication process of Sn‐based perovskite thin film. B) SEM images of the perovskite film prepared through the conventional spin coating method without vacuum treatment. C) SEM images of the perovskite film prepared with vacuum treatment. D) SEM images of the vacuum treated perovskite film containing 2.5% GuaSCN. Specifically, the cross section SEM images corresponding to each sample are shown on the bottom in (B–D).

The perovskite precursor comprises mixtures of stoichiometric phenethylammonium iodide (PEAI), formamidinium iodide (FAI), and SnI_2_ for PEA_2_FA_*n* − 1_Sn_*n*_I_3*n* + 1_ (*n* = 10). We used a one‐step spin‐coating process with vacuum treatment to fabricate perovskite films.^[^ ^43^, ^44^ ^]^ As shown in **Figure** **1**A, after the regular spin‐coating procedure with dripping antisolvent, the wet films were immediately put into an antechamber and pumped for 8 min with a pressure of ≈10^−2^ bar. Subsequently, the samples were taken out and annealed at 70 °C for 10 min to improve their crystallinity. Figure 1B,C shows the morphology and microstructure of the films characterized by a scanning electron microscope (SEM). It is revealed that the perovskite film prepared through the conventional method without vacuum treatment (referred as control) has many cracks and pinholes (Figure 1B), which could lead to shunt path and leakage in the PSCs. By contrast, the film with the vacuum treatment (referred as target‐pristine) shows good coverage after annealing (Figure 1C), which can be ascribed to the fast evaporation of solvent and the quick nuclei formation from the top surface.^[^ ^43^ ^]^ Interesting, the cross‐sectional SEM image reveals that the target‐pristine film contains double‐layer grains, rather than the single‐layer grain structure formed in the control film, which may form a vertically stratified heterostructure (Figure 1C). Then, GuaSCN was introduced as an additive in the precursor solution and the perovskite films were prepared with either the conventional method or the assistance of vacuum treatment. In the former condition, the cracks and pinholes are further enlarged, as observed from Figure S1, Supporting Information, which is detrimental to the device performance. In the latter condition, as shown in Figure 1D, no significant change in the perovskite morphology and grain size can be observed after 2.5% GuaSCN addition and double‐layer grains are still presented (referred as target‐GuaSCN). We found that the optimum addition level of GuaSCN for PSCs is 2.5%, which will be addressed later.

X‐ray diffraction (XRD) measurements were conducted to investigate the film structures. All three films exhibit dominant diffraction peaks at 14.02° and 28.25° assigned to (100) and (200) planes, respectively, of 3D orthorhombic (Amm2) FASnI_3_ structure (Figure S2, Supporting Information). No peak position shift is observed for them, indicating that the 3D perovskite lattice is unchanged at different processing conditions. Additional diffraction peak at around 3.81° for the control film can be assigned to the plane of *n* = 2 quasi‐2D perovskite with double SnI_6_ octahedra layer separated by PEA bilayers.^[^ ^32^ ^]^ This peak slightly moves to lower diffraction angle at 3.56° for both target pristine and GuaSCN containing films and gives rise to an increased lattice constant, probably due to the weakened interaction between the quasi‐2D perovskite interlayers as reported in literature.^[^ ^45^ ^]^

**Figure 2:**
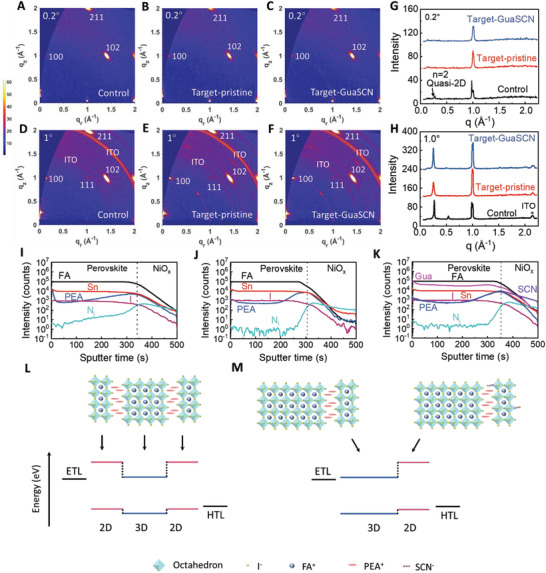
Structure characterizations of perovskite films and schematic energy‐level diagrams in the PSCs. GIWAXS patterns of Sn perovskite films A,D) control, B,E) target‐pristine, C,F) target‐GuaSCN (2.5%) at an incidence angle of (A–C) 0.2° and (D–F) 1° respectively. Intensity plots of the GIWAXS patterns along the *q*_z_ direction at an incidence angle of G) 0.2° and H) 1°. TOF‐SIMS depth profiles of the I) control, J) target‐pristine, and K) target‐GuaSCN (2.5%) film. Schematic crystal structures of the L) control, M) target‐pristine or target‐GuaSCN film, and their energy level diagrams with respect to the HTL and ETL.

Grazing incidence wide‐angle X‐ray scattering (GIWAXS) was further used to characterize the vertical microstructure of the films. Two incidence angles, that is, 0.2° and 1° were selected, which are expected to penetrate < 50 nm and 300–400 nm film depth, respectively, with a view to probing the surface and deep bottom of the films.^[^ ^46^ ^]^ At the incident angle of 0.2°, an intense diffraction spot is observed at *q*_z_ ≈ 1 Å^−1^ for all samples, indicating preferential out‐of‐plane orientation of (100) planes of 3D FASnI_3_ perovskite (**Figure** **2**A–C). According to their intensity plots shown in Figure 2G, the diffraction peak at *q*_z_ ≈ 0.27 Å^−1^ corresponding to the 2D perovskite (*n* = 2) is observed from the control film, while no obvious peak for 2D phase is observed in the target films. At the incident angle of 1°, sharp diffraction spots (at *q*_z_≈ 0.27, 0.54 Å^−1^ for the control film; *q*_z_ ≈ 0.25 Å^−1^ for the target films) emerge for all the samples, as shown in Figure 2D–F,H. The results indicate the presence of *n* = 2 quasi‐2D phase in the whole region of the control film whereas mainly in the middle or bottom of the target films. According to the GIWAXS pattern, the quasi‐2D phases in all three samples have a growth orientation parallel to the substrate. Notably, the signal peaks for quasi‐2D phase in Figure 2H show decreased full width at half maximum with the GuaSCN addition (Table S1, Supporting Information), indicating the improved crystallinity of the quasi‐2D phase induced by GuaSCN.

To characterize the component distribution more precisely in the perovskite films, we performed time‐of‐flight secondary ion mass spectrometry (TOF‐SIMS) depth profile of the films deposited on NiO*_x_*/ITO. As shown in Figure 2I–K, signals of FA^+^, Sn^2+^ and I^−^ in all samples are almost constant throughout the entire perovskite films, indicating their uniform vertical distribution. Interestingly, the distribution of PEA^+^ is mainly located at the bottom of the target films within 80 nm, while it is concentrated on both the surface and the bottom of the control sample, and broadly dispersed from the bottom to the bulk region. This result further confirms broad dispersion of quasi‐2D perovskite in the whole control film while a vertical stratification for the quasi‐2D (down)–3D (top) phase layers in the target films. Previous literature claimed that the difference in the solubility between the 3D and 2D perovskite component could induce their inhomogeneous vertical crystallization.^[^ ^47^ ^]^ As shown in Figure S3, Supporting Information, 3D FASnI_3_ is less soluble than the 2D perovskite in DMF. Hence, the 3D phase is assumed to reach supersaturation and precipitate out earlier than quasi‐2D perovskite in the downward grain growth, leading to the enrichment of quasi‐2D perovskite on the bottom. Moreover, the process conditions (e.g., the solvent drying speed) are critical to the crystallization kinetics. So, it is reasonable to find that ultrafast evaporation of the solvent DMF in vacuum can induce the separation of 3D/2D phases more distinctly, leading to a morphology different from the intercalated 3D and 2D phases in the whole film reported before.^[^ ^48^ ^]^

Relatively strong signals of Gua^+^ and SCN^−^ are observed in the target film with GuaSCN additive, as shown in Figure 2K. The uniformly distributed Gua^+^ is expected to passivate defects by occupying FA^+^ vacancies or interacting with the under‐coordinated I^−^ at the grain boundaries of both the 3D and quasi‐2D perovskite phases.^[^ ^49^ ^]^ SCN^−^ is known to be effective in donating a lone pair electron to Sn^2+^ to inhibit its oxidation.^[^ ^50^ ^]^ Interestingly, the distribution of SCN^−^ closely follows the trend of PEA^+^, implying its enrichment in the bottom quasi‐2D perovskite with a possibility of incorporating into the lattice by replacing I^−^. Based on the above results, the band structure of the PSCs with control and target perovskite films are depicted in Figure 2L,M. The intercalated 2D perovskite phases in the control device may prohibit electron transfer across the whole absorber to the electron transport layer (ETL) while the target devices have a single heterojunction that could be favorable for charge separation under light illumination. The band structure of each layer will be characterized later.

**Figure 3:**
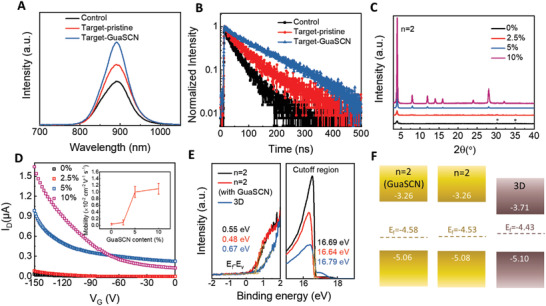
Charge dynamics and electrical characterizations of perovskite films. A) PL spectra of the Sn‐based perovskite films. B) TRPL spectra of the Sn‐based perovskite films. C) XRD patterns of the quasi‐2D PEA_2_FA_1_Sn_2_I_7_ perovskite films processed with different amounts of GuaSCN. D) Transfer curves of the field‐effect transistors prepared by depositing PEA_2_FA_1_Sn_2_I_7_ perovskite films (containing different amounts of GuaSCN) on SiO_2_/Si substrates. Inset: the calculated hole mobility of the corresponding films. E) UPS spectra of the 3D FASnI_3_ and PEA_2_FA_1_Sn_2_I_7_ (w/o or w/10% GuaSCN) perovskite films. F) Energy level diagram of the quasi‐2D perovskite PEA_2_FA_1_Sn_2_I_7_ (w/o or w/10% GuaSCN) and 3D FASnI_3_ films.

To investigate the carrier lifetime of the perovskite films, we conducted steady‐state PL and time‐resolved PL (TRPL) measurements on the films deposited on quartz substrates. A shown in **Figure** **3**A, the PL intensity enhances in turn for the control, target‐pristine and target‐GuaSCN (2.5%) samples. The stronger PL intensity for the target samples indicates reduced defect density and lower non‐radiative recombination rate within the films, especially for the one with GuaSCN additive. Figure 3B shows the TRPL spectra that are fitted with a bi‐exponential decay function and the corresponding decay parameters are listed in Table S2, Supporting Information. The average carrier lifetime of the control sample is 46 ns, which is increased to 68 ns for the target‐pristine film and further prolonged to 148 ns in the target‐GuaSCN film, consistent with the trend in the variation of PL intensities. We note that the carrier lifetime is much longer than those reported for the state‐of‐art Sn‐based perovskite films and almost comparable to lead‐based perovskite films.

As discussed above, GuaSCN additive improves the crystallinity of the bottom quasi‐2D perovskite phase in the target film, as shown in Figure 2H. To reveal such an effect more clearly, we prepared 2D perovskite films (*n* = 2) using precursors of PEA_2_FA_1_Sn_2_I_7_ with additions of GuaSCN. XRD pattern of the pristine perovskite film without GuaSCN indicates that it mainly has a phase with diffraction peaks corresponding to 2D perovskite (*n* = 2) while no peak for other 2D perovskite phases like *n* = 1 can be observed. With the introduction of GuaSCN at different levels from 2.5% to 10%, the peak intensities for 2D perovskite (*n* = 2) are significantly improved, indicating the enhanced crystallinity of the quasi‐2D perovskite (*n* = 2) induced by GuaSCN (Figure 3C). For the precursor of PEA_2_FA_*n* − 1_Sn_*n*_I_3*n* + 1_ (*n* = 10), the addition of 2.5% GuaSCN means that the molar ratio of GuaSCN to PEA^+^ is 12.5:100. Assuming that SCN^−^ is mainly distributed in the bottom quasi‐2D phase as revealed by TOF‐SIMS measurement, we can roughly estimate the amount of SCN^−^ remaining in the bottom quasi‐2D phase to be ≈10%, which is consistent with the optimum condition for achieving high crystallinity of the 2D perovskite (*n* = 2) phase by adding 10% GuaSCN additive in PEA_2_FA_1_Sn_2_I_7_ precursor. This enhanced crystallinity may be ascribed to an oriented growth process for the quasi‐2D phase due to the coordination between SCN^−^ and Sn^2+^.^[^ ^51^ ^]^

To study the electronic property of the quasi‐2D perovskite films (*n* = 2), we prepared field effect transistors by depositing the films on Si/SiO_2_ substrates and measured the drain current (*I*_D_) as a function of gate voltage (*V*_G_), as shown in Figure 3D. All devices show p‐channel and the hole mobility (*μ*) can be determined by:

(1) $$\mu \;=\frac{dI_{D}}{dV_{G}}\;\times \;\frac{L}{W}\times \;\frac{1}{C_{\mathrm{ox}}V_{D}}$$

where *L*, *W*, *C*_ox_, and *V*_D_ are the channel length, width, gate oxide capacitance per unit area, and drain voltage, respectively. The calculated *μ* is around 3.9 × 10^−4^ cm^2^ V^−1^s^−1^ for the pristine film, which is significantly increased to 9.0 × 10^−4^, 1.0 × 10^−2^, and 1.1 × 10^−2^ cm^2^ V^−1^s^−1^ in the films with 2.5%, 5%, and 10% GuaSCN additions, respectively (see the inset in Figure 3D). Hence, the hole mobility in the quasi‐2D perovskite is dramatically improved for 28 times by GuaSCN due to the enhanced crystallinity of the film.

To investigate the energy level diagram of the quasi‐2D/3D heterojunction in the perovskite films, we independently measured the ultraviolet photoelectron spectroscopy (UPS) of quasi‐2D perovskite films (*n* = 2) without and with 10% GuaSCN addition as well as 3D FASnI_3_ (Figure 3E). Combined with the bandgaps extracted from the UV–vis absorption and PL spectra (Figures S4,S5, Supporting Information), the valence band maximum (*E*_V_) and conduction band minimum (*E*_C_) of the films were determined as shown in Figure 3F. It can be found that the pure quasi‐2D perovskite (*n* = 2) film shows a similar valence band level *E*_V_ (−5.08 eV) to 3D FASnI_3_ film (−5.10 eV) while its conduction band level *E*_C_ (−3.26 eV) is much higher than that of FASnI_3_ (−3.71 eV), which can prohibit electron transfer across the quasi‐2D/3D mixed phase shown in Figure 2L. Notably, GuaSCN doping has little effect on the band structure of the quasi‐2D film. Hence, we can obtain the bandstructures of the PSCs based on pristine, target, and target‐GuaSCN perovskite films shown in Figure 2L,M. For the devices with vacuum treatment, the heterojunction can prohibit carrier diffusion to the opposite directions, enhance charge separation, and alleviate carrier recombination in the devices.

**Figure 4:**
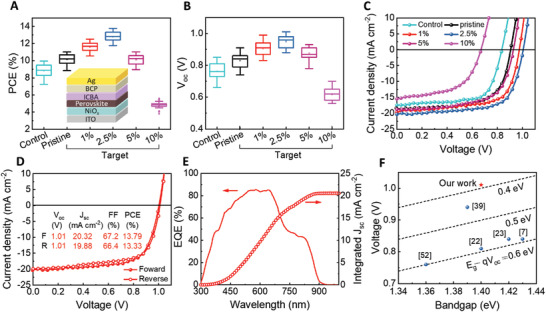
Device structure and performance. A) PCE statistics of 25 control or target (pristine or containing 1%, 2.5%, 5%, and 10% GuaSCN) PSCs. Inset: the device structure of PSCs. B) *V*_OC_ statistics of 25 control or target (pristine or containing 1%, 2.5%, 5%, and 10% GuaSCN) PSCs. C) *J–V* curves of the control or target (pristine or containing 1%, 2.5%, 5%, and 10% GuaSCN) PSCs. D) *J–V* curves of the champion target‐GuaSCN (2.5%) PSC under different scan directions. E) The EQE spectrum and integrated *J*_SC_ of the champion target‐GuaSCN (2.5%) PSC. F) *V*_OC_ loss of Sn‐based PSCs reported in this work and previous literature.

PSCs with a configuration of ITO/NiO*_x_*/perovskite/indene‐C60 bisadduct (ICBA)/bathocuproine (BCP)/Ag were fabricated, as shown in the inset in **Figure** **4**A. The control PSCs based on PEA_2_FA_*n* − 1_Sn_*n*_I_3*n* + 1_ (*n* = 10) perovskite exhibit relatively low PCE with large deviation, as shown in Figure 4A. The inferior performance can be ascribed to the broad distribution of quasi‐2D phase in the control film, which may prohibit electron transfer from the perovskite film to the ETL as illustrated in Figure 2L. Additionally, the cracks and pinholes in the control film also can induce severe leakage in the devices, and the device PCE gets even worse for the film containing GuaSCN additive processed w/o vacuum treatment (Figure S6, Supporting Information). By contrast, the target devices with vacuum treatment exhibit a higher average PCE with narrower distribution due to the compact morphology and the formation of a vertical quasi‐2D/3D heterojunction in the films. Then, we optimized the concentration of GuaSCN in the target devices. It can be seen that the average device PCE gradually increases with the increase of GuaSCN molar ratio from 0% to 2.5% and then decreases with more addition of GuaSCN from 5% to 10%, and the statistics of *V*_OC_, short‐circuit current density (*J*_SC_), and fill factor (FF) basically follow the same trend (Figure 4B; Figure S7, Supporting Information). Figure 4C presents the best performing *J–V* curves of the PSCs processed at different conditions. The control PSC exhibits a PCE of 9.95%, *V*_OC_ of 0.83 V, *J*_SC_ of 17.40 mA cm^−2^, and FF of 68.9%. For the target‐pristine PSC, it shows improved PCE to 11.02%, with significantly improved *V*_OC_ to 0.91 V and *J*_SC_ to18.37 mA cm^−2^. The target‐GuaSCN (2.5%) processed PSC yields the best PCE of 13.79%, with a remarkably enhanced *J*_SC_ to 20.32 mA cm^−2^ and *V*_OC_ to 1.01 V. It is notable that the defect passivation effect of GuaSCN and the 2D/3D heterojunction formed in target‐GuaSCN perovskite film synergistically lead to the significantly improved *J*_SC_ and *V*_OC_, owing to the reduced carrier recombination in the device. Benefiting from this, the *J–V* curves of the champion target‐GuaSCN device in forward scan and reverse scan almost overlap with negligible hysteresis (Figure 4D). The external quantum efficiency (EQE) spectrum of the champion device was measured as shown in Figure 4E, from which the integrated *J*_SC_ (20.56 mA cm^−2^) is close to that obtained from the *J–V* curve. Notably, the *V*_OC_ loss is merely 0.39 V at a bandgap of 1.40 eV (see Figure S8, Supporting Information) for the absorber, which is comparable with Pb‐based PSCs (Figure 4F).^[^ ^7^, ^22^, ^23^, ^39^, ^52^ ^]^ The highest *V*_OC_ of the device indicates the best effect of defect passivation by the quasi‐2D and GuaSCN. Moreover, the target‐GuaSCN PSCs exhibit good long‐term stability in N_2_ atmosphere. As shown in Figure S9, Supporting Information, the PCE only drops for about 10% of the initial value after 1200 h.

*V*_OC_ of a PSC is closely related to its *J*_SC_ and dark saturation current density (*J*_0_) described by $V_{OC}=\frac{nKT}{q}\ln\left(\frac{J_{\mathrm{SC}}}{J_{0}}+1\right)$. Figure S10, Supporting Information shows the dark *J–V* curves of the target PSCs, from which *J*_0_ can be extracted by fitting the curves with the Shockley diode equation (Figure S11, Supporting Information). The target‐GuaSCN PSC possesses a much lower *J*_0_ (3.38 × 10^−17 ^mA cm^−2^) than that of the target‐pristine PSC (4.50 × 10^−15^ mA cm^−2^), indicating the reduced carrier generation rate in the former. Hence, the enhancement of *V*_OC_ observed in the GuaSCN containing PSC is consistent with the deceased *J*_0_ of the device.

We plotted the curves of the *V*_OC_ versus light intensity as shown in Figure S12, Supporting Information. The ideality factors of 1.41 and 1.34 are obtained for the target‐pristine and target‐GuaSCN PSCs via extracting the slope of the plots, which reflect the type of recombination in the devices. The value between 1 and 2 indicates that recombination occurs via trap‐assisted Shockley–Read–Hall recombination. The smaller slope of the devices doped with GuaSCN reveals less interfacial or bulk trap recombination. All the results indicate that high *V*_OC_ can be achieved when the density of traps in the Sn‐based PSCs is decreased to a low level.

**Figure 5:**
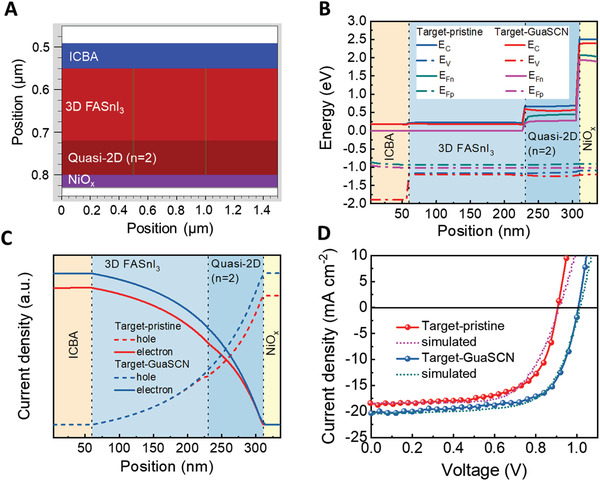
Device simulation results for the target‐pristine or target‐GuaSCN PSCs by the software Silvaco. A) Schematic PSC structure with vertical 2D/3D heterojunction used for simulation. B) The energy levels including the conduction band (*E*_C_), the valence band (*E*_V_), hole (*E*_Fp_), and electron (*E*_Fn_) quasi‐Fermi levels across the whole devices under the open‐circuit condition. C) The distribution of hole and electron carrier densities across the PSCs under AM 1.5 G illumination without bias voltage (short‐circuit condition). D) Simulated *J–V* curves (dash lines) together with the experimental results (solid lines) of the PSCs with the vacuum treatment.

To further clarify the effect of GuaSCN on the performance improvement of target PSCs, we did device simulation using a commercial software Silvaco.^[^ ^53^, ^54^ ^]^ A planar quasi‐2D (80 nm)/3D (170 nm) heterostructure, according to our SEM image, is created in the PSCs as schematically shown in **Figure** **5**A, and the other parameters for simulation are listed in Table S3, Supporting Information. The major difference in the two devices is the carrier mobilities in the 2D perovskite phase, which increase from 3.9 × 10^−4^ to 0.011 cm^2^ V^−1^s^−1^ after the introduction of GuaSCN. As shown in Figure 5B, a cascade energy level alignment is observed in both PSCs under the open circuit condition. In comparison with the target‐pristine PSC, the target‐GuaSCN PSC exhibits a larger gap between the electron quasi‐Fermi level (*E*_Fn_) close to the cathode and the hole quasi‐Fermi level (*E*_Fp_) close to the anode, corresponding to a higher *V*_OC_ of the device. In addition, Figure 5C shows the distribution of carrier concentrations in the devices under AM 1.5 G illumination without bias. Carrier concentrations are higher in the target‐GuaSCN PSC due to the improved carrier mobility and lifetime induced by GuaSCN. Figure 5D presents the simulated *J–V* curves of the PSCs, which are almost comparable to our experimental results. Therefore, GuaSCN plays an important role in not only passivating defects in the perovskite film but also enhancing the crystallinity of the 2D perovskite phase with improved carrier mobility that can facilitate carrier transport in the PSCs.

## 3 Conclusion

In this work, we develope a vacuum‐assisted one‐step spin coating method that enables the formation of a vertically stratified 2D/3D heterojunction in Sn‐based perovskite films with full coverage. We find that the band structure of the heterojunction is favorable for carrier separation and transfer across the junction. Moreover, we demonstrate the role of GuaSCN additive in tuning the electronic properties of the heterojunction, including constructing conducting channels for hole transfer in 2D layer, and reducing trap‐assisted recombination loss in the whole film. As a result, we obtain a PCE of 13.79% from the champion Sn‐based PSCs and achieve the maximum *V*_OC_ of 1.01 V, which is the highest value ever reported for Sn‐based PSCs. Interestingly, the *V*_OC_ loss of 0.39 V observed in the devices is comparable to that of Pb‐based counterparts, indicating that the major drawback of Sn‐based PSCs in their low open circuit voltages has been overcome by our special techniques.

## 4 Experimental Section

### Materials

FAI and PEAI were purchased from Greatcell Solar Materials Ltd. GuaSCN, SnF_2_, and BCP were purchased from Sigma Aldrich. Nickel(II) nitrate hexahydrate (Ni(NO_3_)_2_·6H_2_O) and SnI_2_ (99.999%) were purchased from Alfa Aesar. ICBA was purchased from 1‐Material. NiO*_x_* nanocrystals were synthesized according to previous work.

### Solar Cell Fabrication

Cleaned ITO glass substrates were treated with O_2_ plasma for 6 min in priority. Then NiO*_x_* solution (6.5 mg mL^−1^, dispersed in deionized water) was spin‐coated on the substrates at 4000 rpm for 30 s and annealed at 150 °C for 20 min in the air. Afterward, the NiO*_x_* substrates were transferred into a N_2_‐filled glovebox. Sn‐based perovskite precursor solution was prepared by dissolving a 2:9:10 stoichiometric ratio of PEAI, FAI, and SnI_2_ together with 10 mol% SnF_2_ in mixed DMF/DMSO solvents (v:v = 4:1) at a concentration of 0.9 m. For GuaSCN modification, GuaSCN at a molar ratio of 1%, 2.5%, 5%, and 10% to SnI_2_ was added to the above solution. The precursor solution was spin‐coated at 2000 rpm for 3 s and 5000 rpm for 40 s, and chlorobenzene (CB) was dripped as the anti‐solvent at 30th second during the second spinning process. The wet perovskite films were immediately put into an antechamber and pumped for 8 min with a pressure of ≈10^−2^ bar. Subsequently, the samples were taken out and annealed at 70 ℃ for 10 min to induce better crystallization. Next, ICBA (20 mg mL^−1^, dissolved in CB) was spin coated at 1500 rpm for 30 s, and annealed at 70 ℃ for 5 min. BCP (0.5 mg mL^−1^ in IPA) was then spin‐coated at 5000 rpm for 30 s. Finally, 100 nm Ag was thermally deposited on top of BCP via an Angstrom deposition system.

### Field‐Effect Transistor Fabrication

The Si/SiO_2_ (300 nm) substrates were ultrasonically cleaned by deionized water/acetone/isopropyl alcohol in sequence for 5 min and dried under a stream of nitrogen gas. Cr (10 nm)/Au (100 nm) electrodes with a channel length of 4 µm and a channel width of 800 µm were patterned via photolithography and magnetron sputtering on the Si/SiO_2_ substrates. The quasi‐2D perovskite thin films were deposited on top of the substrates using the one‐step spinning process with dripping anti‐solvent as previously described.

### Characterization

SEM images were characterized by a field emission SEM (Tescan MAIA3). XRD measurements were performed on a diffractometer (Rigaku Smartlab) using Cu K*α* radiation (*λ* = 1.54 Å). GIWAXS were conducted on a Xeuss 2.0 SAXS/WAXS laboratory beamline using a Cu X‐ray source (8.05 keV, 1.54 Å) and a Pilatus3R 300K detector. Two grazing‐incidence angles, that is, 0.2° and 1.0° were used. UV–vis spectra were measured by a UV–vis–NIR spectrometer (Perkin Elmer). Steady‐state PL and TRPL were measured using Edinburgh FLS920 fluorescence spectrophotometer with an excitation wavelength of 488 nm. It was noted that for PL and TRPL characterizations, the perovskite films were deposited on quartz substrates, and then encapsulated by covering another quartz on top of the films to avoid oxidation in the air. UPS was performed on a VG ESCALAB 220i‐XL ultrahigh vacuum surface analysis system equipped with a He‐discharge lamp providing He‐I photons at 21.22 eV. Notably, the perovskite films for UPS measurements were loaded on the sample holder in the glovebox, then taken out and quickly transferred to the vacuum chamber with only several seconds’ exposure in the air. TOF‐SIMS V (ION‐TOF GmbH) was used for depth profiling of the elements distribution in the perovskite films. The Bi_3_^+^ primary ion beam (25 keV, 0.3 pA) was used for analysis and scanned at an area of 50 × 50 µm^2^. The sputtering was accomplished with the Cs^+^ ion beam (1 keV, 5 nA) over an area of 200 × 200 µm^2^.

Photocurrent density‐voltage (*J–V*) curves of PSCs were measured using a Keithley 2400 source meter under the illumination of AM 1.5 G, 100 mW cm^−2^ solar simulator (Newport 91160). The active area of the PSCs was 4.8 mm^2^. The EQE of the PSCs was measured using an EQE system equipped with a xenon lamp (Newport 66902), monochromator (Newport 74125), and a Si detector (Newport 71675_71580).

### Electrical Measurements for the Field‐Effect Transistors

The devices were measured using a semiconductor parameter analyzer (Keithley 4200‐SCS, Solon, Ohio, USA) in a nitrogen‐filled glovebox. The *I*_D_ versus *V*_G_ curves were measured at the *V*_D_ of 0.5 V.

## Conflict of Interest

The authors declare no conflict of interest.

## Supporting information

Supporting Information

## Data Availability

The data that support the findings of this study are available in the Supporting Information of this article.
