# Supplementary material for: High Open Circuit Voltage Over 1 V Achieved in Tin‐Based Perovskite Solar Cells with a 2D/3D Vertical Heterojunction
Source: Adv Sci (Weinh). 2022 Apr 22;9(18):2200242. doi: 10.1002/advs.202200242 (PMC9218751; doi:10.1002/advs.202200242)
Supplement: Supplementary file 1 — Supporting Information [file ADVS-9-2200242-s001.pdf]

Supporting Information

High open circuit voltage over 1 V achieved in tin-based perovskite solar cells with a 2D/3D vertical heterojunction

*Tianyue Wang, Hok-Leung Loi, Jiupeng Cao, Zhaotong Qin, Zhiqiang Guan, Yang Xu, Haiyang Cheng, Mitch Guijun Li, Chun-Sing Lee, Xinhui Lu, and Feng Yan**

Dr. T. Y. Wang, H. L. Loi, Dr. J. P. Cao, H. Y. Cheng, Prof. F. Yan*
Department of Applied Physics, The Hong Kong Polytechnic University, Hung Hom, Kowloon, Hong Kong SAR, P.R. China
E-mail: apafyan@polyu.edu.hk

Prof. F. Yan*
Research Institute of Intelligent Wearable Systems, The Hong Kong Polytechnic University, Hung Hom, Kowloon, Hong Kong, P. R. China.

Z. T. Qin, Prof. X. H. Lu
Department of Physics, The Chinese University of Hong Kong, Shatin, Hong Kong SAR, P.R. China

Dr. Z. Q. Guan, Prof. C.S. Lee
Center of Super-Diamond and Advanced Films (COSDAF), Department of Chemistry, City University of Hong Kong, Kowloon Tong, Hong Kong SAR, P.R. China

Y. Xu, Prof. M. Li
Division of Integrative Systems and Design, Department of Electronic and Computer Engineering, The Hong Kong University of Science and Technology, Clear Water Bay, Kowloon, Hong Kong SAR, P.R.China

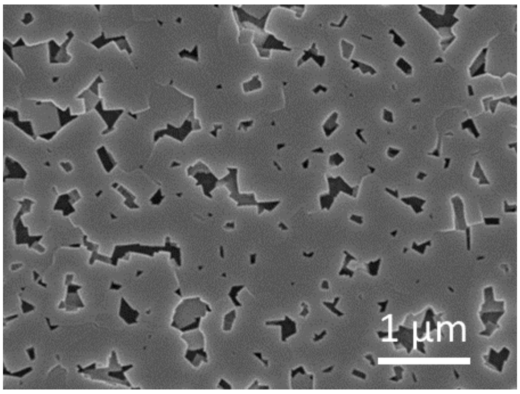

Figure S1. The SEM image of the perovskite film containing 2.5% GuaSCN prepared through the conventional spin coating method.

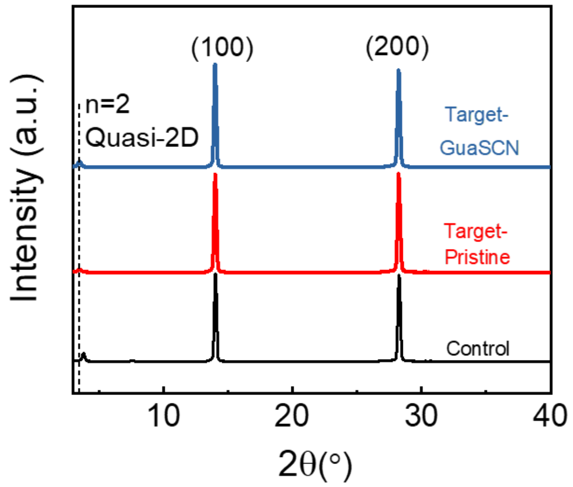

Figure S2. XRD patterns of the control, target-pristine, target-GuaSCN (2.5%) Sn-based perovskite films.

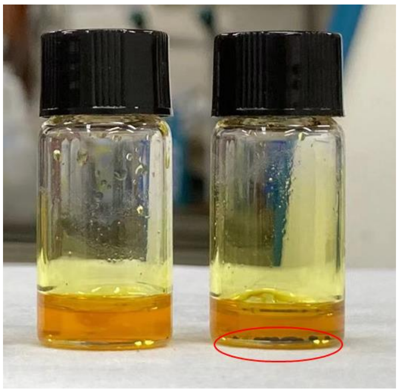

Figure S3. Photographs of $PEA_2FA_1Sn_2I_7$ (2.25M) (left) and $FASnI_3$ (4.5M) (right) dissolved in DMF solution.

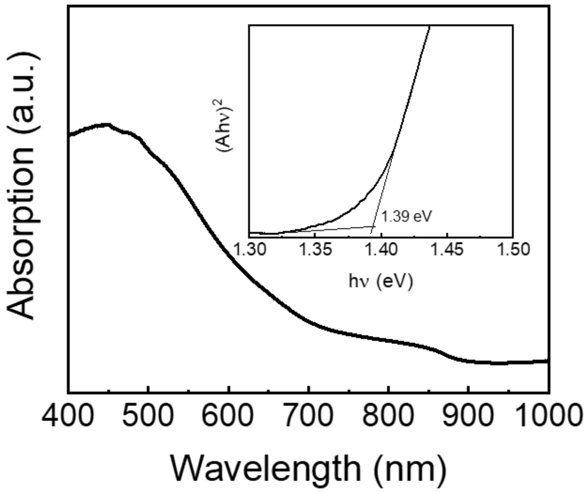

Figure S4. The UV-vis spectrum and Tauc plot of a 3D $FASnI_3$ perovskite film.

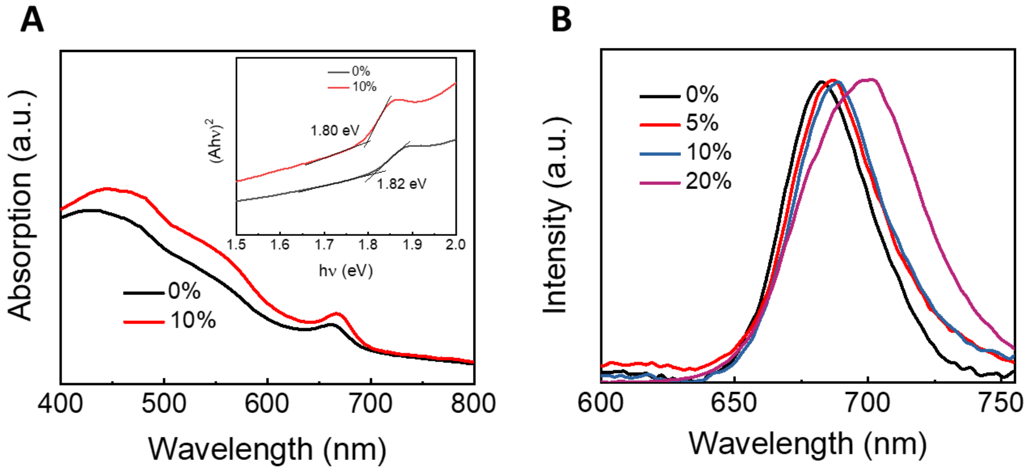

Figure S5. (A) UV-vis spectra and Tauc plots of $PEA_2FA_1Sn_2I_7$ perovskite films with 0% and 10% GuaSCN. (B) PL spectra of $PEA_2FA_1Sn_2I_7$ perovskite films processed with varied amounts of GuaSCN additive.

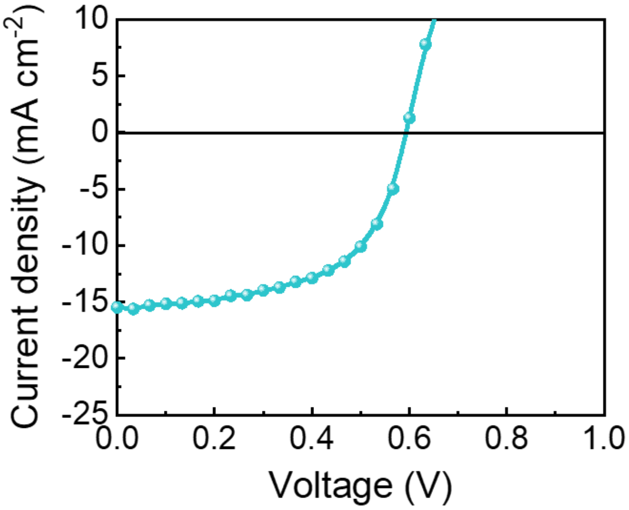

Figure S6. The J-V curve for the PSC containing 2.5% GuaSCN additive processed w/o vacuum treatment.

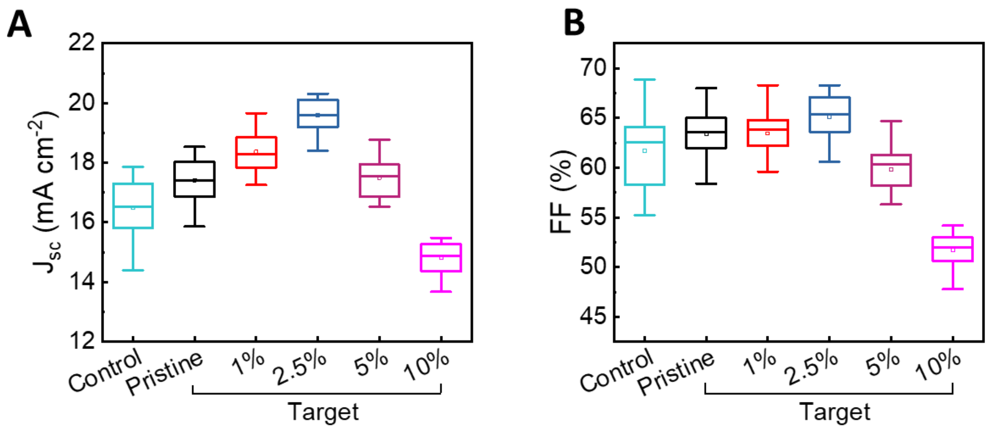

Figure S7. (A) J_{sc} and (B) FF statistics of 25 control or target (pristine or containing 1%, 2.5%, 5%, 10% GuaSCN) PSCs.

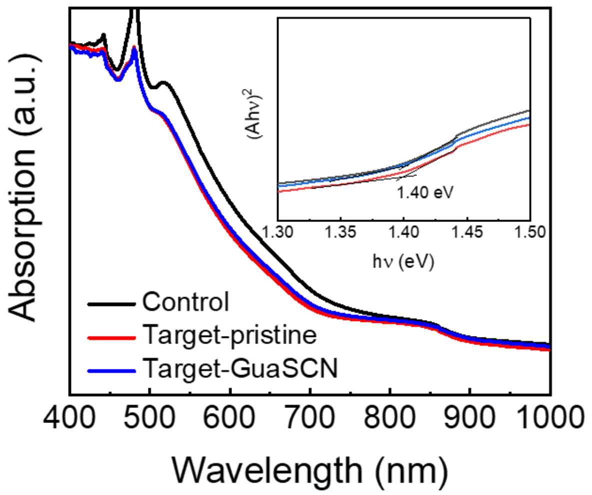

Figure S8. UV-vis spectra and Tauc plots of the control, target-pristine, target-GuaSCN Sn-based perovskite films.

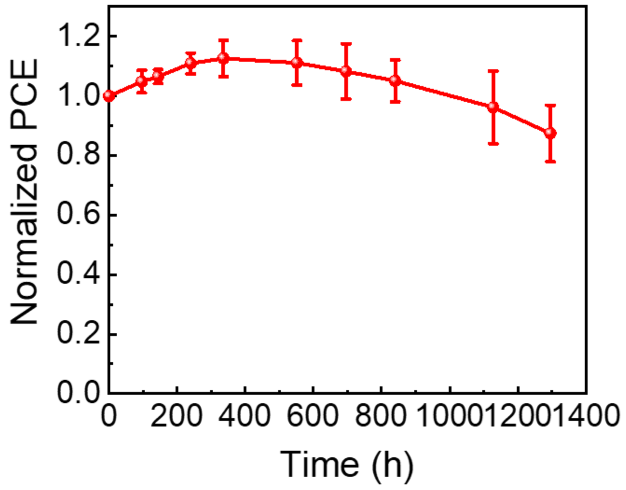

Figure S9. Long term stability of the target-GuaSCN (2.5%) devices stored in N_2 filled glovebox (The error was derived from 3 devices).

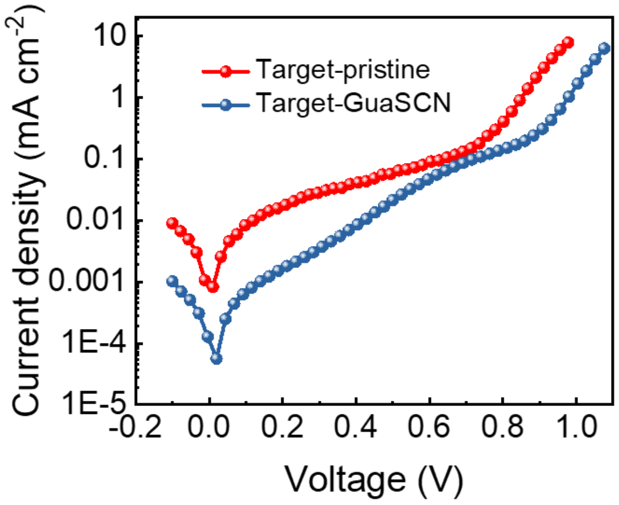

Figure S10. Dark J-V curves of the target-pristine and target-GuaSCN PSCs.

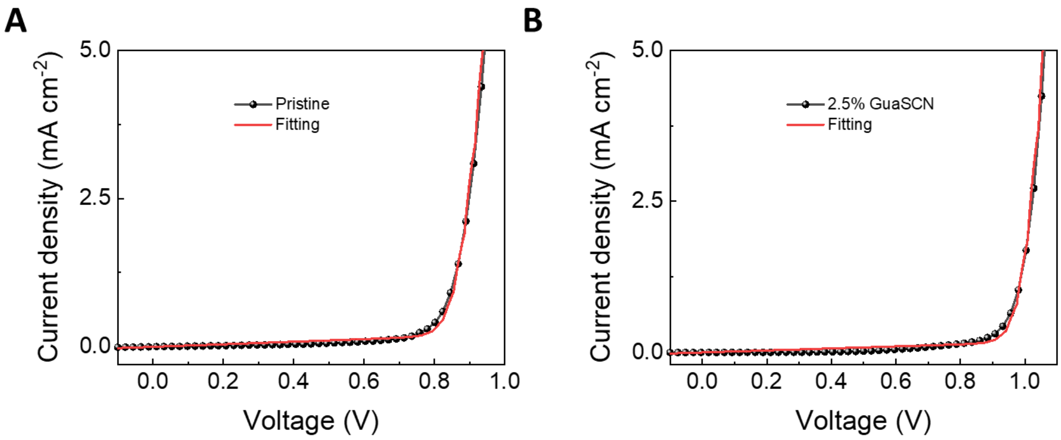

Figure S11. Fitting of the dark J-V curves of the target-pristine and target-GuaSCN PSCs with the Shockley diode equation.

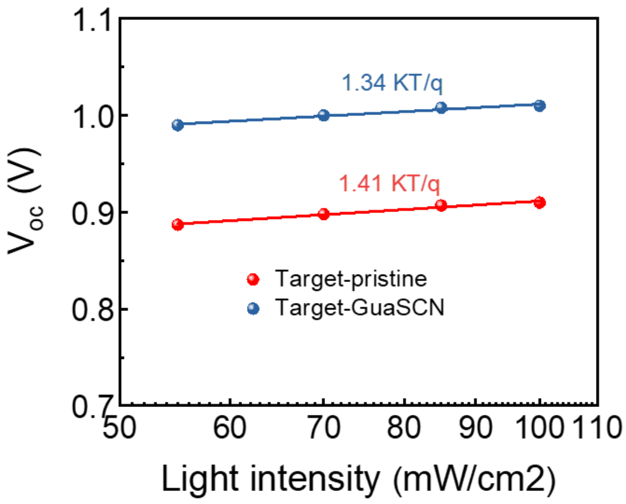

Figure S12. Light intensity dependence of V_{oc} for the target-pristine and target-GuaSCN PSCs.

Table S1. The full width at half maximum (FWHM) of different peaks shown in Figure 2H.

Sample	FWHM at q≈0.25 Å^{-1}	FWHM at q≈1 Å^{-1}
Target-pristine	0.073	0.033
Target-GuaSCN	0.026	0.029

Table S2. Fitted results of TRPL curves shown in Figure 3B.

**Samples**	**A_1**	**τ_1 (ns)**	**A_2**	**τ_2 (ns)**	**τ_{avg} (ns)**
Control	0.26	8	0.74	48	46
target-pristine	0.102	14	0.898	69	68
target-GuaSCN	0.1	21	0.9	150	148

Note: The bi-exponential decay function $Y = A_1\exp(-t/\tau_1) + A_2\exp(-t/\tau_2)$ is used for fitting, where $\tau_{avg} = \frac{\sum_{i=1}^{n} A_i\tau_i^2}{\sum_{i=1}^{n} A_i\tau_i}$

Table S3. Summarized simulation parameters of the PSCs

**Parameters and units**	**Symbols**	**NiO_x**	**$PEA_2FASn_2I_7$ (with GuaSCN)**	**$FASnI_3$**	**ICBA**
Thickness (nm)	L	30	80	170	60
Band Gap at 300 K (eV)	E_g	3.6	1.82 (1.80)	1.39	2.07[1]
Electron Affinity (eV)	χ	1.46[2]	3.26 (3.26)	3.71	3.74[1]
Electron density of states at 300 K (#/cm^3)	N_C	2.5×10^{20}	2.5×10^{20}	2.5×10^{20}	2.5×10^{20}
Hole density of states at 300 K (#/cm^3)	N_V	2.5×10^{20}	2.5×10^{20}	2.5×10^{20}	2.5×10^{20}
Hole mobility (cm^2 V^{-1} s^{-1})	μ_h	0.003	3.9×10^{-4} (0.011)	0.1	0.01
Electron mobility (cm^2 V^{-1} s^{-1})	μ_e	0.003	3.9×10^{-4} (0.011)	0.1	0.01[3]
Relative dielectric constant	ε_r	11.7[4]	5.7	5.7[5]	3

References：

[1] B. B. Yu, Z. Chen, Y. Zhu, Y. Wang, B. Han, G. Chen, X. Zhang, Z. Du, Z. He, *Adv. Mater.* **2021**, 2102055.

[2] T. Wang, F. Zheng, G. Tang, J. Cao, P. You, J. Zhao, F. Yan, *Adv. Sci.* **2021**, 2004315.

[3] E. Orgiu, M. A. Squillaci, W. Rekab, K. Börjesson, F. Liscio, L. Zhang, P. Samorì, *Chemical Communications* **2015**, *51*, 5414.

[4] F. Roccaforte, G. Greco, P. Fiorenza, V. Raineri, G. Malandrino, R. Lo Nigro, *Applied Physics Letters* **2012**, *100*, 063511.

[5] C. Ran, W. Gao, J. Li, J. Xi, L. Li, J. Dai, Y. Yang, X. Gao, H. Dong, B. Jiao, I. Spanopoulos, C. D. Malliakas, X. Hou, M. G. Kanatzidis, Z. Wu, *Joule* **2019**, *3*, 3072.
